# Early Diagnosis and Targeted Therapy in SLC39A8-Congenital Disorder of Glycosylation: A Case Report From Bulgaria

**DOI:** 10.7759/cureus.101202

**Published:** 2026-01-09

**Authors:** Valentina Varbanova, Genoveva Tacheva, Teodora Paneva, Marina Krasteva, Dimitar Stamatov, Ivan Litvinenko

**Affiliations:** 1 Pediatrics, Medical University of Sofia, Sofia, BGR; 2 Pediatric Neurology, SBAL Children’s Hospital “Prof. Dr. Ivan Mitev”, Sofia, BGR

**Keywords:** congenital disorder of glycosylation, dystonia, global developmental delay, hypotonia, manganese transportation, slc39a8-cdg

## Abstract

SLC39A8-congenital disorder of glycosylation (SLC39A8-CDG) is a rare autosomal recessive metabolic disease of manganese transport, leading to defective glycosylation and mitochondrial dysfunction. An eight-month-old male infant with severe hypotonia, developmental delay, and dystonic episodes was initially misdiagnosed as epilepsy. Genetic testing identified a homozygous pathogenic variant in the *SLC39A8 *gene, and biochemical analysis confirmed low manganese levels. Upon initiation of oral manganese sulfate therapy, the patient demonstrated significant clinical improvement, including the achievement of new motor milestones. To our knowledge, this is the first documented case in Bulgaria. This case underscores the importance of early genetic diagnosis and targeted metabolic treatment in altering the clinical trajectory of SLC39A8-CDG. Timely recognition allows for intervention in a disorder that, despite its rarity, has a modifiable course and potential for meaningful developmental gains.

## Introduction

Congenital disorders of glycosylation (CDG) comprise a diverse and expanding group of inherited metabolic diseases caused by defects in the synthesis, attachment, or modification of glycans. These processes are fundamental to the proper function of glycoproteins and glycolipids, particularly within the central nervous system [1]. Among the rarer subtypes is SLC39A8-CDG, a disorder of trace metal transport that disrupts both glycosylation and mitochondrial function [2,3].

Тhe *SLC39A8* gene encodes the manganese (Mn) transporter ZIP8, responsible for the cellular uptake of Mn²⁺- a critical cofactor for several glycosyltransferases in the Golgi apparatus, as well as for the mitochondrial superoxide dismutase (Mn-SOD) [4]. Inadequate levels of intracellular Mn lead to defective glycan maturation and oxidative stress, contributing to a wide spectrum of neurological manifestations, including developmental delay, hypotonia, dystonia, and cerebellar atrophy [3,5].

## Case presentation

**Figure 1:**
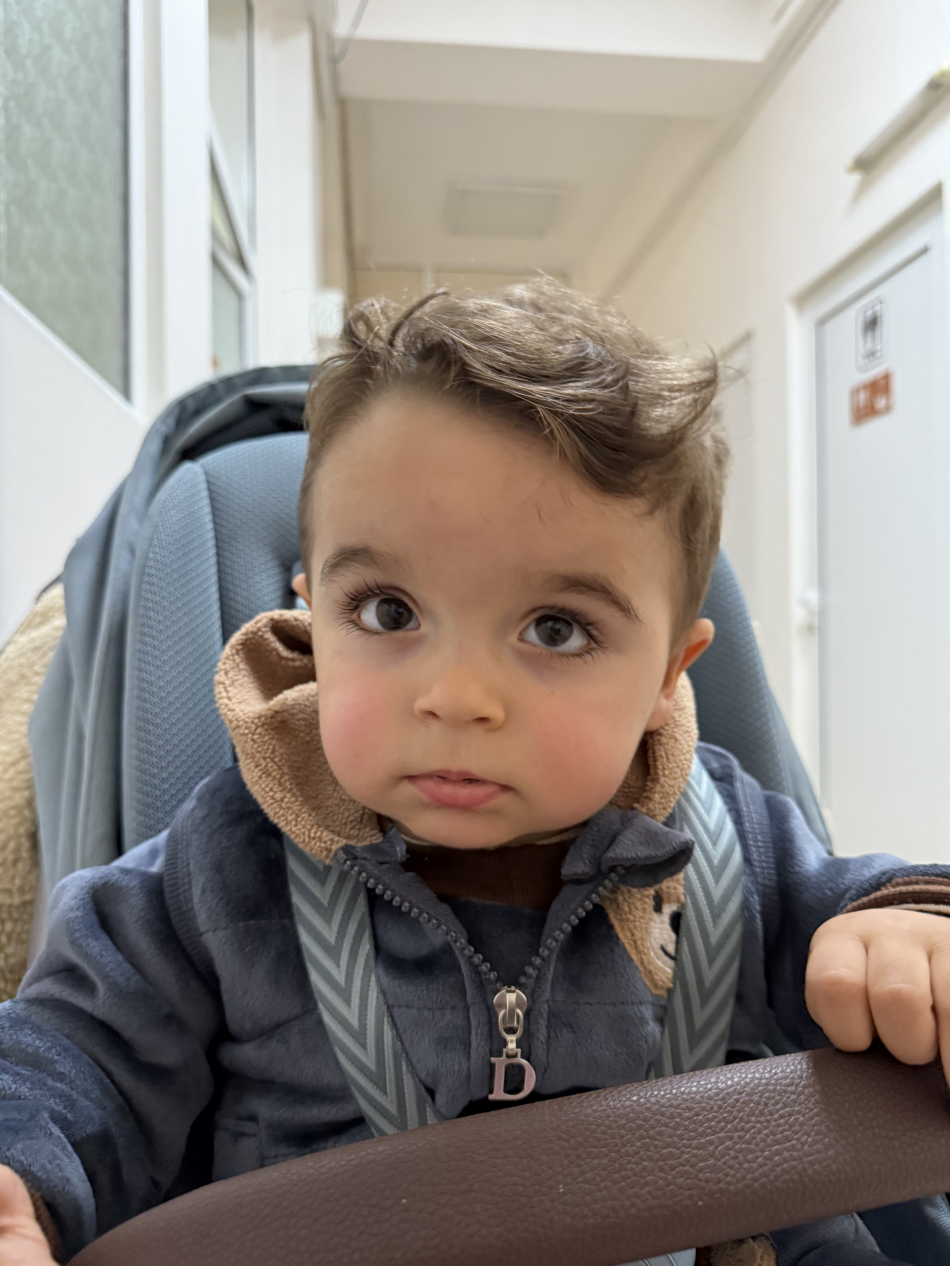
Dysmorphic features of the patient. The photo was obtained and is published with the signed and informed consent of the parents.

An eight-month-old male child with an uneventful perinatal and neonatal history, the first child of non-consanguineous parents, developed stereotyped episodes initially interpreted as focal epileptic seizures, characterized by ocular deviation and sustained gaze. These were misinterpreted as focal epileptic seizures. On examination, mild facial dysmorphism was noted, including hypertelorism, broad forehead, and thin lips (Figure 1). Neurodevelopmental assessment demonstrated severe global developmental delay with absent gross motor milestones as well as impaired visual fixation and minimal social engagement.

Cranial nerve examination identified convergent strabismus. Further neurological assessment revealed generalised muscle hypotonia, predominantly for the axial musculature, significantly increased deep tendon reflexes with expanded reflexogenic zones, and positive Babinski and Rossolimo signs bilaterally. 

The clinical course was further complicated by recurrent episodes of vomiting and poor appetite lasting two to three days, without evidence of infection. During this period, intravenous glucose infusions were administered. Outside these periods, the child was exclusively fed orally. For a period of time, no weight gain was registered, and even a decline in his weight was observed. At eight months, the patient weighed 8.7 kg (+0.82 Standard Deviation Score (SDS) relative to the WHO standards), decreasing to 7.6 kg (-1.18 SDS) at nine months and remaining unchanged at 12 months (-2.00 SDS), followed by catch-up growth to 12 kg (+1.16 SDS) at 18 months.

**Figure 2:**
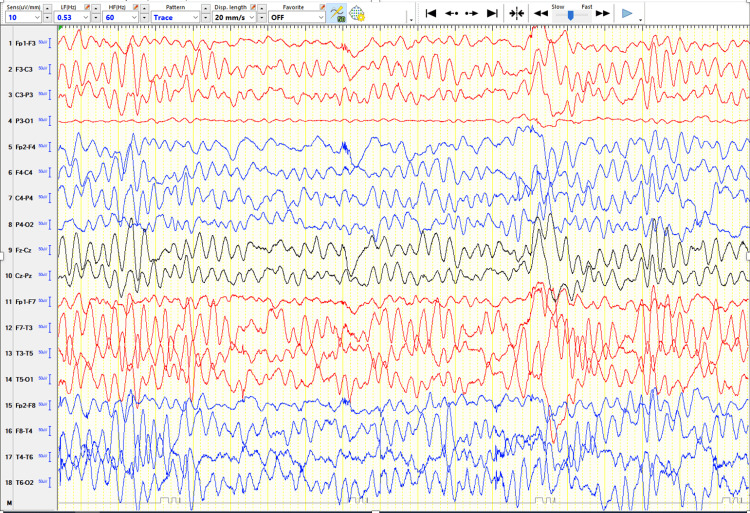
EEG recorded in the awake state at eight months of age, displayed in a longitudinal bipolar (double banana) montage using the international 10–20 system, showing diffuse high-amplitude slow wave activity and no definite epileptiform discharges, consistent with global cerebral dysfunction.

**Figure 3:**
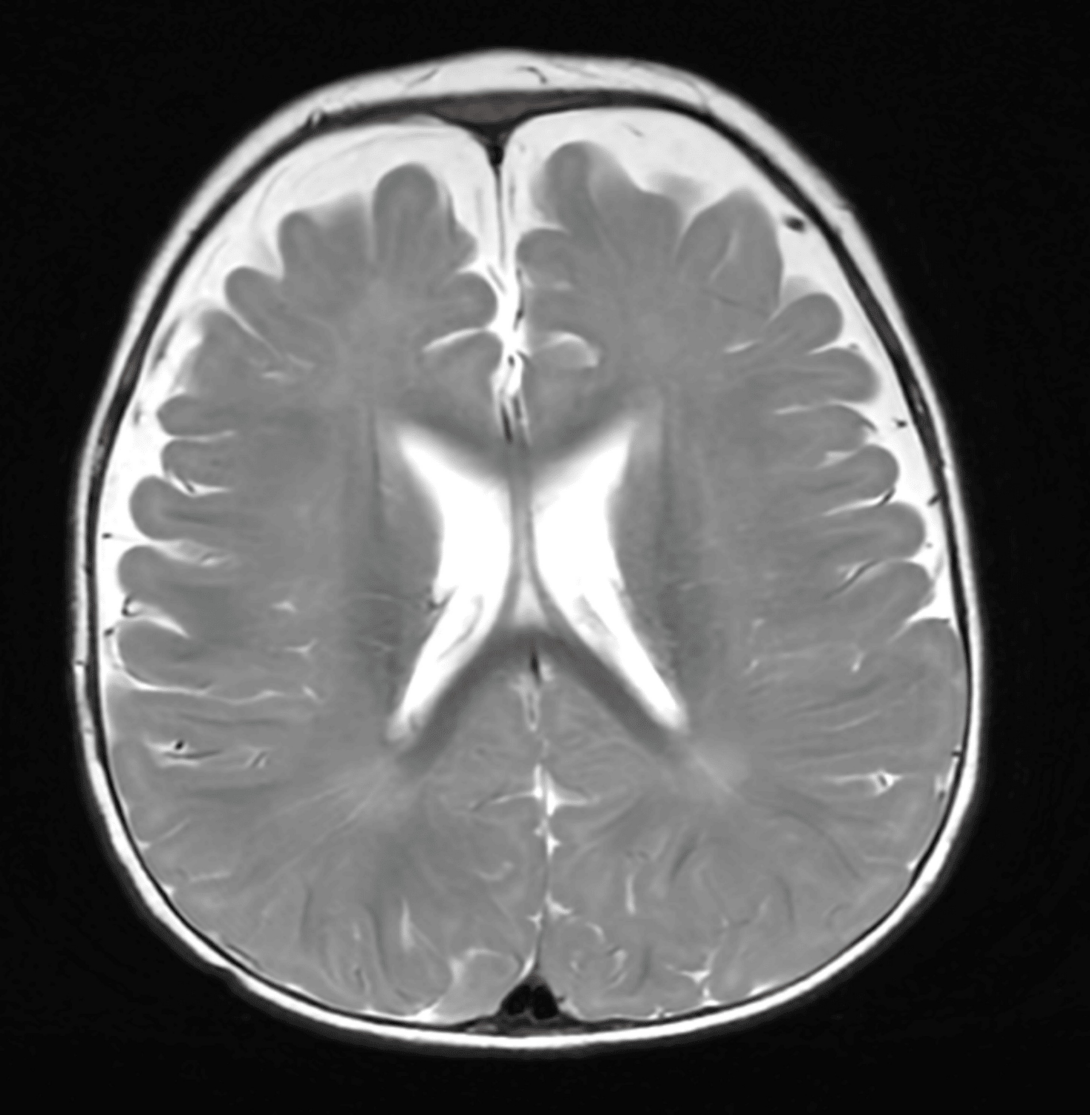
Brain MRI (Axial T2-weighted fast spin-echo) performed at eight months of age demonstrating diffuse, symmetric widening of the extra-axial CSF spaces over the frontal and parietal convexities, with preserved cortical morphology and normal ventricular size, consistent with BESS BESS: benign enlargement of subarachnoid spaces; CSF: cerebrospinal fluid

**Figure 4:**
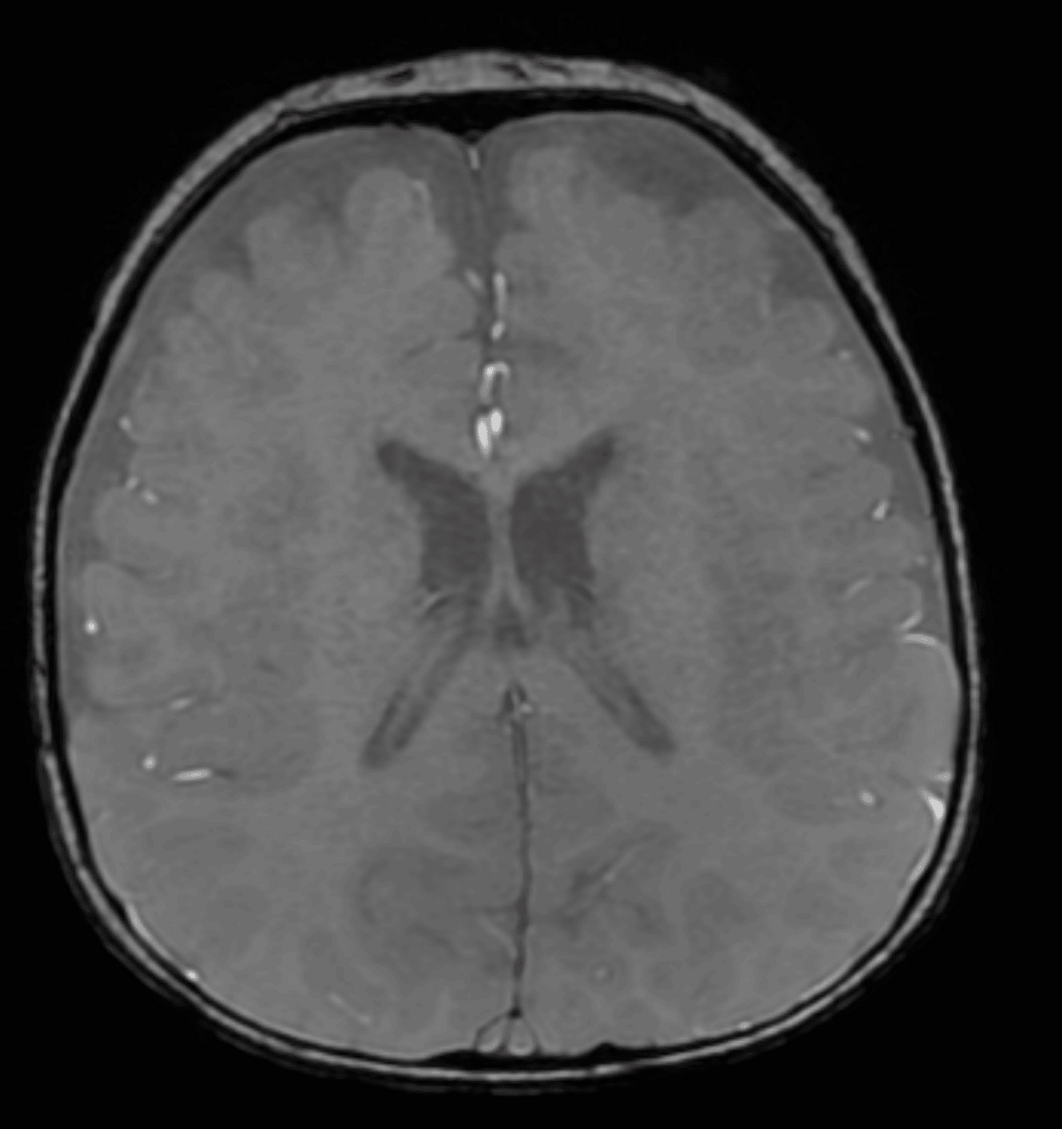
Brain MRI (Axial SWAN) performed at eight months of age showing prominent extra-axial CSF spaces without evidence of intracranial hemorrhage or abnormal susceptibility, supporting the diagnosis of BESS SWAN: susceptibility-weighted angiography; CSF: cerebrospinal fluid; BESS: benign enlargement of subarachnoid spaces

Electroencephalography (EEG) showed bilateral high-amplitude slow wave activity in the occipital regions, as in metabolic diseases (Figure 2). Brain MRI demonstrated benign enlargement of the subarachnoid spaces (BESS), without other structural abnormalities (Figures 3, 4).

Although the initial clinical impression favoured focal epilepsy, a movement disorder was also considered early in the differential diagnosis, prompting a next-generation sequencing (NGS) panel targeting dystonia-associated genes. During follow-up with another pediatric neurologist, the stereotyped episodes were captured during video-EEG monitoring and showed no associated paroxysmal EEG activity. Based on these findings, the events were reclassified as dystonic rather than epileptic in origin. Initial treatment with levetiracetam was started under the working diagnosis of epilepsy. Following diagnostic clarification, treatment was later tapered.

NGS identified a homozygous likely pathogenic variant in the *SLC39A8* gene: NM_001135146.1:c.112G>C, p.(Gly38Arg), consistent with SLC39A8-CDG. Both parents were heterozygous carriers of the same variant. Once the diagnosis was clear, follow-up investigations were conducted, measuring serum manganese levels, which revealed a low-normal concentration of 2.6 µg/L (reference range: 0-15 µg/L). Serial monitoring over the following months demonstrated persistently reduced serum manganese levels. Serum Mn level, measured in a reference laboratory in Germany, was 4.2 µg/L (reference range: 6-11 µg/L), which corresponds more closely to values reported in the literature for patients with SLC39A8-CDG. The last result was obtained while the patient was receiving ongoing low-dose therapy with manganese sulfate (MnSO₄) (4 mg/kg/day). 

Targeted metabolic therapy with oral MnSO₄ was initiated at a dose of 6.25 mg/kg/day. After initiation of supplementation, deterioration of hypotonia was noted, and the dosage was slowly tapered down to 3 mg/kg/day. At his last check-up at age 18 months, an increase in dosage was conducted (4 mg/kg/day). The patient demonstrated significant developmental improvement, including independent sitting, standing up by himself, smiling, and making eye contact.

Informed consent

Written and signed informed consent for publication of the patient's clinical information and images was obtained from the patient's legal guardian.

## Discussion

This report describes the first genetically confirmed case of SLC39A8-CDG in Bulgaria and highlights the diagnostic challenge posed by its early neurological presentation, as well as the potential for meaningful clinical improvement following targeted Mn supplementation. Recognition of the underlying metabolic and genetic etiology enabled initiation of disease-specific treatment, resulting in clear developmental gains. This case, therefore, underscores the clinical relevance of early genetic testing in infants with unexplained neurodevelopmental impairment and movement disorders, particularly in potentially treatable metabolic conditions such as SLC39A8-CDG.

CDG is a heterogeneous group of monogenic diseases with predominantly autosomal recessive inheritance and multisystemic involvement. Defects in glycosylation could be classified in four categories: (i) N-linked glycosylation, (ii) O-linked glycosylation, (iii) combined N- and O-linked/multiple glycosylation, and (iv) lipid and glycosylphosphatidylinositol (GPI) anchor biosynthesis defects [1].

N-glycosylation is a complex process involving the addition of carbohydrate chains to asparagine residues within Asn-X-Ser/Thr motifs in the endoplasmic reticulum with subsequent modification in the Golgi apparatus [4]. Defects at any stage of this pathway can result in congenital disorders of glycosylation. PMM2-CDG, the most common CDG subtype, is caused by mutations in the *PMM2* gene, impairing the conversion of mannose-6-phosphate to mannose-1-phosphate in GDP-mannose synthesis. Most patients are compound heterozygotes, with p.Arg141His being the most frequent variant among Europeans. Genotype-phenotype correlations have been described. Specific genotypes were associated with either visceral or non-visceral types of symptoms [2].

O-linked glycosylation encompasses the process of adding carbohydrate chains to the amino acids serine, threonine, and hydroxylysine residues of proteins by glycosyltransferases in the Golgi apparatus [1]. GPI (glycosylphosphatidylinositol) anchors are glycolipid structures synthesised in a stepwise manner within the endoplasmic reticulum and subsequently modified in the Golgi apparatus. They serve as membrane anchors for numerous cell surface proteins, mediating a wide range of cellular functions. Genetic defects affecting enzymes involved in GPI-anchor biosynthesis cause inherited disorders collectively referred to as GPI biosynthesis defects. These conditions are named alphabetically based on the order of gene discovery rather than the specific biosynthetic step. Most are inherited in an autosomal recessive pattern except for *PIGA*-related disorders, which are X-linked [1].

Among the various types of CDG, SLC39A8-CDG represents a distinct and rare subtype that does not result directly from defects in glycosylation enzymes themselves, but from impaired metal ion transport, specifically, Mn, an essential cofactor for several glycosyltransferases. The first reported case of SLC39A8-CDG was in 2015 [1]. It is inherited in an autosomal recessive pattern. SLC39A8-CDG determines a defect in the transport protein ZIP8, which is localised in different cellular organelles such as mitochondria and the Golgi apparatus. In the Golgi, Mn is necessary as a cofactor of glycosyltransferases responsible for attaching galactose to glycolipids and glycoproteins. Mn is also of high importance to Mn-dependent mitochondrial superoxide dismutase. Low levels of Mn, therefore, lead to hypogalactosylation of lipids and proteins as well as impaired mitochondrial function [5].

According to ClinVar, more than 18 distinct *SLC39A8* variants associated with SLC39A8-CDG have been reported to date [6]. Of these, nine are classified as variants of uncertain significance, three as likely pathogenic, three as pathogenic, one as pathogenic/likely pathogenic, one as benign, and one has conflicting interpretations of pathogenicity. Regarding variant type, the majority are missense variants, followed by nonsense and frameshift variants; in addition, 3′UTR and intronic variants have also been reported. The missense variant identified in our patient is classified as pathogenic/likely pathogenic according to the American College of Medical Genetics and Genomics (ACMG) criteria [7].

As previously described, SLC39A8-CDG is a multisystem disorder with variable clinical expression. Reported manifestations can be broadly grouped into neurological, gastroenterological, ophthalmological, and dysmorphic features. Neurological symptoms include severe hypotonia, dystonia, epilepsy, developmental delay, and ataxia. Feeding difficulties are common and may result in failure to thrive. The most frequently reported ophthalmological abnormality is strabismus. Dysmorphic features include a broad forehead, excess hair around the mouth and chin, an upturned nose, thin lips, a smooth philtrum, and skeletal abnormalities, with some individuals later developing scoliosis [8].

Across published reports comprising a total of 17 genetically confirmed patients, developmental and intellectual disability were present in all cases: hypotonia in 16/17, dystonia or dyskinesia in 7/17, epilepsy in 9/17, feeding difficulties in 7/17, dysmorphism in 5/17, skeletal abnormalities in 7/17, and strabismus in 11/17 [8-11]. Symptom onset most commonly occurred in infancy, typically between three and five months of age. In the present case, developmental delay and severe hypotonia were evident within the first months of life, consistent with previously reported cases; however, medical attention was first sought at eight months of age following the appearance of paroxysmal dystonic episodes. The patient exhibited feeding difficulties with failure to thrive, strabismus, dystonia, and characteristic facial dysmorphism, aligning closely with the core clinical spectrum of SLC39A8-CDG.

MRI studies of patients with SLC39A8-CDG show variable findings. The most common trait is cerebellar atrophy. It is considered a specific sign of severe CDG. Other common features include cerebral atrophy [5] and basal ganglia lesions resembling Leigh syndrome. Although cerebellar atrophy is frequently reported, no neuroimaging finding is sufficiently sensitive or specific to establish the diagnosis of SLC39A8-CDG. A diagnosis is made based on multisystemic involvement and typical biochemical changes. Confirmation by genetic testing is required: whole exome sequencing (WES), whole genome sequencing (WGS), multigene panel, or targeted gene panel. Regarding the paraclinical investigation, the hallmark of the disease is a low serum level of Mn [10], which was also registered in our patient. Patients with SLC39A8 deficiency present with an abnormal transferrin isoelectric focusing (Tf IEF) pattern, characteristic of a type II CDG [10,12]. The Tf IEF shows increased levels of di-sialo and asialo transferrin isoforms, reflecting the defective terminal glycosylation due to impaired addition of galactose and sialic acid residues. 

Management of patients with SLC39A8-CDG requires a multidisciplinary approach. Supportive treatment includes physical therapy and rehabilitation, orthopaedic interventions when necessary. If epilepsy occurred, antiepileptic treatment should be initiated. Growth failure due to feeding difficulties should be corrected by feeding therapy or placing a gastrostomy. Hearing aids may be helpful if hearing is impaired. 

Specific treatment of SLC39A8-CDG targets correction of the systemic Mn deficiency, which disrupts key Mn-dependent enzymes. The preferred approach is oral MnSO₄ supplementation, typically initiated at low doses (≈10 mg MnSO₄/day) and gradually titrated up, often reaching 15-20 mg/kg/day to achieve normalisation of transferrin glycosylation and Mn‑dependent enzyme activity [11]. Treatment outcomes include restoration of β‑1,4‑galactosyltransferase activity, improvеment of mitochondrial superoxide dismutase function, normalisation of transferrin isoelectric focusing (IEF) profiles, and significant clinical benefits such as improved motor skills, muscle tone, hearing, seizure control, and swallowing [12]. In some patients, oral D‑galactose (up to 3.75 g/kg/day), occasionally combined with uridine, has been used to further enhance glycosylation. Galactose should be initiated after the maximum dosage of Mn is achieved in order to avoid masking manganese efficacy signals [13]. Throughout therapy, regular monitoring of transferrin glycosylation, whole‑blood Mn levels, and periodic brain MRI (every one to two years) is essential to balance efficacy and prevent Mn accumulation or neurotoxicity [12].

The long-term outlook for individuals with SLC39A8-CDG remains uncertain, largely because most reported cases involve patients at a young age who are yet to be observed. Prognosis can vary significantly depending on the genotype-phenotype correlation, the age of onset, and the age of treatment initiation [8].

## Conclusions

SLC39A8-CDG is a rare and treatable CDG that can present with neurological and systemic symptoms. Early recognition through genetic testing, metabolic screening, and imaging is essential for timely diagnosis.

This case highlights the effectiveness of manganese supplementation in improving clinical outcomes, particularly in developmental milestones. Prognosis varies depending on the severity of symptoms and the timing of treatment, underscoring the importance of early intervention and multidisciplinary care to optimise patient outcomes.
